# Ex Vivo Irritation Evaluation of a Novel Brimonidine Nanoemulsion Using the Hen's Egg Test on Chorioallantoic Membrane (HET-CAM)

**DOI:** 10.7759/cureus.68280

**Published:** 2024-08-31

**Authors:** Shahla S Smail

**Affiliations:** 1 Department of Pharmaceutics, College of Pharmacy, Hawler Medical University, Erbil, IRQ

**Keywords:** brimonidine, propylene glycol, het-cam, irritation, nanoemuslion

## Abstract

Introduction: The hen's egg test on chorioallantoic membrane (HET-CAM) assay is a cost-effective and well-validated, non-animal-based *ex vivo* method for evaluating the irritant potential and eye toxicity of substances. A colloidal dispersion of a surfactant and a cosurfactant with a nanosize range is called a nanoemulsion (NE), which is formed by mixing immiscible liquids and stabilized by surfactants. Patients with glaucoma are commonly prescribed Brimonidine (BR), an alpha-2 adrenergic agonist, to lower their intraocular pressure.

Materials and methods: In this study, surfactant-cosurfactant blends were prepared by mixing Tween 80 (surfactant) and propylene glycol (cosurfactant) in a 4:1(v/v) ratio. Triacetin served as the oil phase, while deionized water was used as the aqueous phase. Using the drop method, a range of NE formulations (F1, F2, F3, FB1, FB2, and FB3) were developed and subsequently evaluated for their potential to irritate, and then the results were compared to those of a commercially available BR eye drop formulation.

Results: According to the average cumulative HET-CAM test scores (IS), from excipients, propylene glycol caused moderate irritation by causing slight damage to blood vessels. The formulations FB1 and F1 were found to have the highest level of irritation among other formulations in the investigation, recording 1.05 ±0.07 and 1.2 ±0.10, respectively. All other NE formulations exhibited non-irritating potential, as confirmed by the HET-CAM test, and were comparable to the marketed BR eye drop formulation.

Conclusion: The NE formulations created for BR were determined to be safe and non-irritating. The findings indicate that the prepared NE could be a beneficial solution for addressing problems with conventional eye drops and delivering BR effectively to the eyes.

## Introduction

Composed of nanometer-sized droplets, nanoemulsions (NEs) are colloidal systems that combine two immiscible liquids, oil and water [1]. To guarantee long-term stability, an appropriate emulsifier or a blend of emulsifiers is added. These amphiphilic emulsifiers are molecules that exhibit surface-active properties. Consequently, they can move, bind, and rearrange in interfacial regions, resulting in the formation of small, stable droplets with reduced interfacial tension [2]. Typically, NEs are either oil-in-water (O/W) or water-in-oil (W/O), with O/W being the more prevalent option [3].

NEs stand out due to their remarkably small droplets, exceptional stability, and expansive surface area. Their exceptional attributes make them well-suited for the development of delivery systems that can encapsulate, protect, modulate the release, and enhance the availability of various lipophilic bioactive compounds in industries including pharmaceuticals, cosmetics, biotechnology, and food [1]. The main difference between NEs and MEs (microemulsions) is the lack of thermodynamic stability in NEs. Furthermore, the droplet size distribution of NEs remains unchanged even when diluted with water. Determining the final characteristics and stability of the system relies on the NE fabrication technique [4].

With the formula 5-bromo-N-quinoxalin-6-amine, Brimonidine (BR) is a chemical compound that acts as a highly selective α2-adrenoreceptor agonist. Intraocular pressure (IOP) is lowered as it reduces aqueous humor production and improves its drainage through the uveoscleral pathway. The main purpose of using BR is to treat open-angle glaucoma and ocular hypertension [5,6].

In 2018, BR had over three million prescriptions in the United States, making it one of the 157th most commonly prescribed medications. Its neuroprotective properties against retinal ganglion necrosis are well-documented. However, the effectiveness of BR in treating retinal vascular disorders is not well-established [6].

Testing must be done to assess the potential irritation caused by a drug, chemical, or pesticide before it can be authorized and marketed [7]. Regulatory bodies in various European countries, such as Germany, Netherlands, UK, and France, have endorsed the hen's egg test on chorioallantoic membrane (HET-CAM) assay as a complete substitute for animal testing when assessing severe irritants. The HET-CAM method decreases animal distress, is faster, cheaper, and simpler compared to the Draize test and utilizes a scoring system that is more consistent and less subjective [8].

Nevertheless, the HET-CAM model is not suitable for testing substances that are sticky, pigmented, or water-insoluble. *In vivo*, irritation studies can be replaced by the HET-CAM. This offers a promising alternative to animal experiments, especially in countries where chick embryos are not considered live animals until a specific stage of development or hatching (before 17 embryo development days), eliminating legal authorization concerns when sacrificing the embryos. With its complex vascular system and textural characteristics, the HET-CAM is a perfect choice for conducting experiments on tumor growth, drug delivery, chemotherapy, radiotherapy, cancer metastasis, and other biological phenomena [9-12].

Kim et al. compared in vitro models, such as the HET-CAM assay, with clinical data to investigate the correlation. The HET-CAM test and human patch test for anionic and amphoteric surfactants showed a robust positive association [13]. In the same way, Löffler et al. combined the HET-CAM test with non-invasive imaging techniques (PET or MRI) to pre-select during radiopharmaceutical development, decreasing the need for animal experiments, and achieving comparable results to mouse model studies [12].

This study aimed to create a preservative-free, non-irritating ocular drug delivery system for BR in the form of a controlled-release NE to treat glaucoma. Additionally, it examined the eye irritancy of the BR formulations using the HET-CAM assay and compared the results with those of commercially available BR eye drops.

## Materials and methods

Materials

A crucial ingredient Brimonidine (BR) was supplied by TCI Chemicals (Japan). ACROS Organics (USA) provided additional materials such as triacetin, Tween 80, and propylene glycol. Sigma-Aldrich (Germany) provided sodium hydroxide (NaOH) and sodium chloride (NaCl). AllerganÒ (Ireland) provided BR eye drops with a concentration of 0.2%. The fertilized eggs for the HET-CAM test were obtained from a local farm (Iraq). The remaining chemicals used were of analytical grade.

Methods

Preparation of the BR NE

A mixture of surfactant (Tween 80) and selected cosurfactant (propylene glycol) was used to prepare the Smix solution, with a ratio of 4:1. Following that, the oil (triacetin) and Smix ratio were mixed extensively in glass vials using different volume ratios from 1:9 to 9:1. Next, the aqueous phase (deionized water) was introduced to the oil phase, causing a gradual dilution of the oil phase by the water phase. The NE formulation that exhibited no significant aggregation was selected through visual evaluation [14].

Then the drug BR was incorporated into the optimized formulations at a concentration of 0.2% (w/v). Visual assessment was conducted on the optimized NEs to evaluate clarity, transparency, phase separation, and birefringence. Distinguishing between emulsions and NEs is possible through visual assessment. Emulsions are characterized by their turbidity, whereas NEs are transparent. The system's transparency was confirmed by measuring its percent transmittance at 600 nm using a UV-visible spectrophotometer (Jenway 7315 Spectrophotometer, Bibby Scientific Ltd, UK).

*Ex vivo* ocular irritation testing through the HET-CAM 

An automatic rotary incubator (96 Egg Poultry Brooder, Pet Scene, China) with controlled temperature (37 ^o ^C) and relative humidity (45-65%) was used for incubating fertilized, freshly collected (not more than seven days old) White Leghorn eggs weighing between 50 and 60 g.

To avoid the embryo from sticking to the eggshell, the eggs were rotated by hand several times a day for three days. By the third day, the eggs had been sprayed with methylated spirit, cracked open, and relocated to a growth chamber consisting of a glass beaker and cellophane membrane. On day 10, the eggs underwent inspection to confirm the embryos' viability, the chorioallantoic membrane's (CAM's) integrity, and the yolk. Any eggs with deceased embryos or ruptured yolks were disposed of, and the rest were incubated longer. Eggs that showed a noticeable vascular network on the CAM were chosen for testing.

Following that, the test substances were administered onto the CAM surface, using 200 microliters at ambient temperature. The evaluation included the following experimental groups: 0.9% w/v NaCl solution (negative control), and 0.1 M NaOH solution (positive control). Examination of the membrane and blood vessels occurred at designated time points (0, 0.5, 2, 5 min), with scores being recorded for irritation-induced hemorrhage (vessel bleeding), lysis (vessel integration), and coagulation extra and intravascular protein denaturation). Each endpoint's appearance time was carefully monitored and recorded in seconds. The equation used to calculate the irritation score (IS) was as follows:

 IS = 5 x (301 - H)/ 300} + 7 x (301 -L)/300 + [9 x (301 - C)/30 … (1)

The IS was determined by measuring the time, in seconds, for hemorrhage (H), hyperemia (L), and coagulation (C) to occur. The irritant responses of individuals were represented as the mean and relative standard deviation percentage (RSD%). The observed time-dependent effects were used to assign numerical scores to each of the three response types as shown in Table 1.

**Table 1:** Numerical scores for irritant responses over time. Source: [14]

Score	Effect		
Time (min)	Hyperemia	Hemorrhage	Coagulation
0.5	5	7	9
2.0	3	5	7
5.0	1	3	5

In addition, the damage to the hen's egg-CAM was classified based on the calculated irritation score. An irritation score less than 0.9 was considered non-irritant, a score between 1 and 4.9 was classified as slight irritant, a score between 5 and 8.9 was considered moderate irritant, and a score greater than 9 was classified as strong irritant [7,8,15,16].

Statistical analysis

The student t-test was used to compare the means of the two groups. On the other hand, the one-way analysis of variance (ANOVA) was used to compare the means of three or more groups. This test allows us to determine if there is a significant difference between the means of these groups. To perform the statistical analysis, the data was input into GraphPad Prism (GraphPad Software, San Diego, CA), a software commonly used for statistical analysis. Mean and standard deviation were calculated for each group. A p-value less than 0.05 was considered statistically significant.

## Results

The composition of each of the three different formulations of BR NEs is displayed in Table 2.

**Table 2:** Brimonidine nanoemulsion composition.

Formulations		Water %	S mix blend % (4:1)	Oil %
BR loaded	Blank	Deionized water	Tween 80: propylene glycol	Triacetin
(mg/ml)				
FB1	F1	15	76.5	8.5
FB2	F2	15	51	34
FB3	F3	60	36	4

The NE preparations were observed to be clear, transparent, and without significant aggregations. The transmittance they showed was over 98% (99.3 ±0.062 %), suggesting a one-phase system that is isotropic.

**Figure 1:**
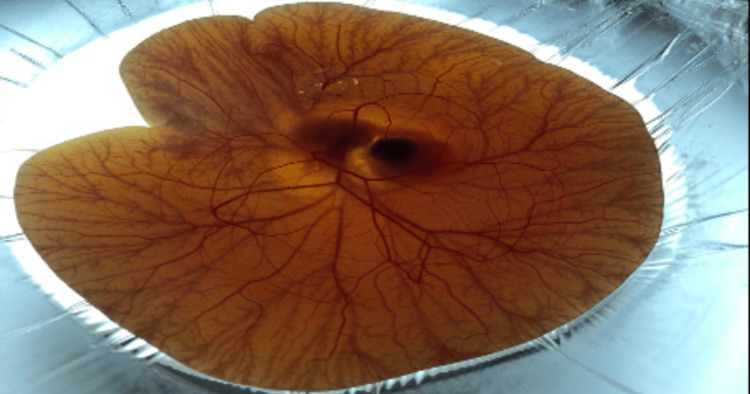
Image of the CAM's surface vascular responses at day 10. CAM: Chorioallantoic membrane

Figure 1 shows the vascular responses of CAM's surface which resembles vascular mucosal tissue in the human eye.

**Figure 2:**
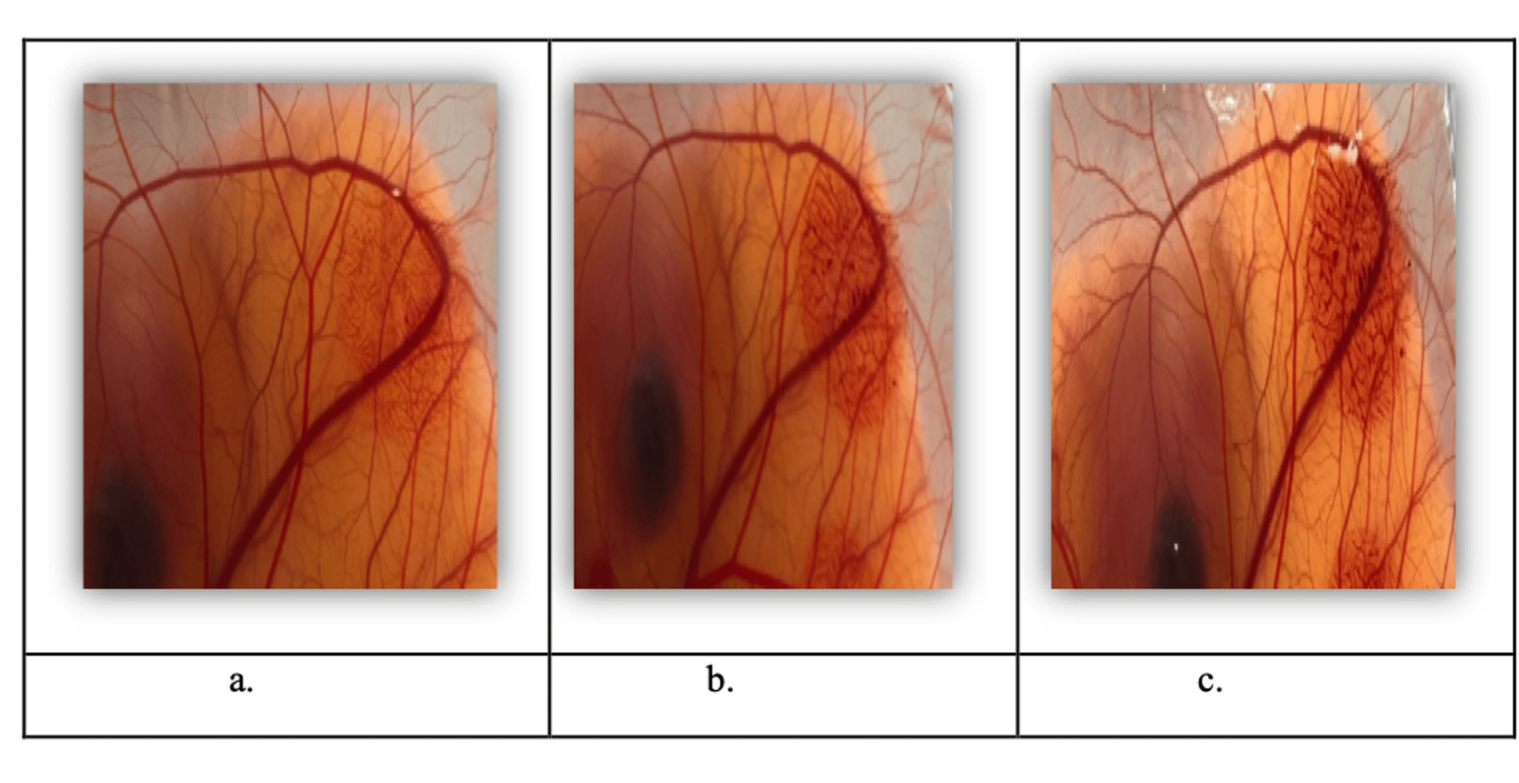
The CAM's surface vascular responses to a positive control substance (0.1 M NaOH): a. 0.5 minutes, b. 2 minutes, and c. 5 minutes post-application. CAM: Chorioallantoic membrane

Figures 2a-2c demonstrate a significant irritation response on the CAM's surface, such as complete blood vessel lysis, clotting, and coagulation, upon exposure to the positive control substance (0.1 M NaOH). Nevertheless, the CAM surface showed no changes when exposed to the negative control substance NaCI (0.9 % w/v).

The average cumulative HET-CAM test scores for the optimized NE formulations (F1, F2, and F3), BR-loaded formulations (FB1, FB2, and FB3), the marketed BR eye drops, and the individual components (tween 80, propylene glycol, and triacetin) are shown in Table 3.

**Table 3:** The HET-CAM test yielded cumulative irritation scores and irritancy classification. The data are presented in this table as mean +_SD, n=3. HET-CAM: Hen's egg test on chorioallantoic membrane

Test substance	IS	Classification
NaCI 0.9 % w/v	0 ± 0.00	Non-irritant
NaOH 0.1 M	17.8 ± 0.57	Extreme irritant
Tween 80	0.39 ± 0.03	Non-irritant
Propylene Glycol	5.1 ± 0.05	Moderate-irritant
Triacetin	0.29 ± 0.04	Non-irritant
F 1	1.05 ± 0.07	Slight-irritant
F 2	0.30 ± 0.05	Non-irritant
F 3	0.10 ± 0.05	Non-irritant
FB1	1.2 ± 0.10	Slight-irritant
FB2	0.10 ± 0.05	Non-irritant
FB3	0.08 ± 0.02	Non-irritant
Marketed BR eye drop	0.09 ± 0.08	Non-irritant

**Figure 3:**
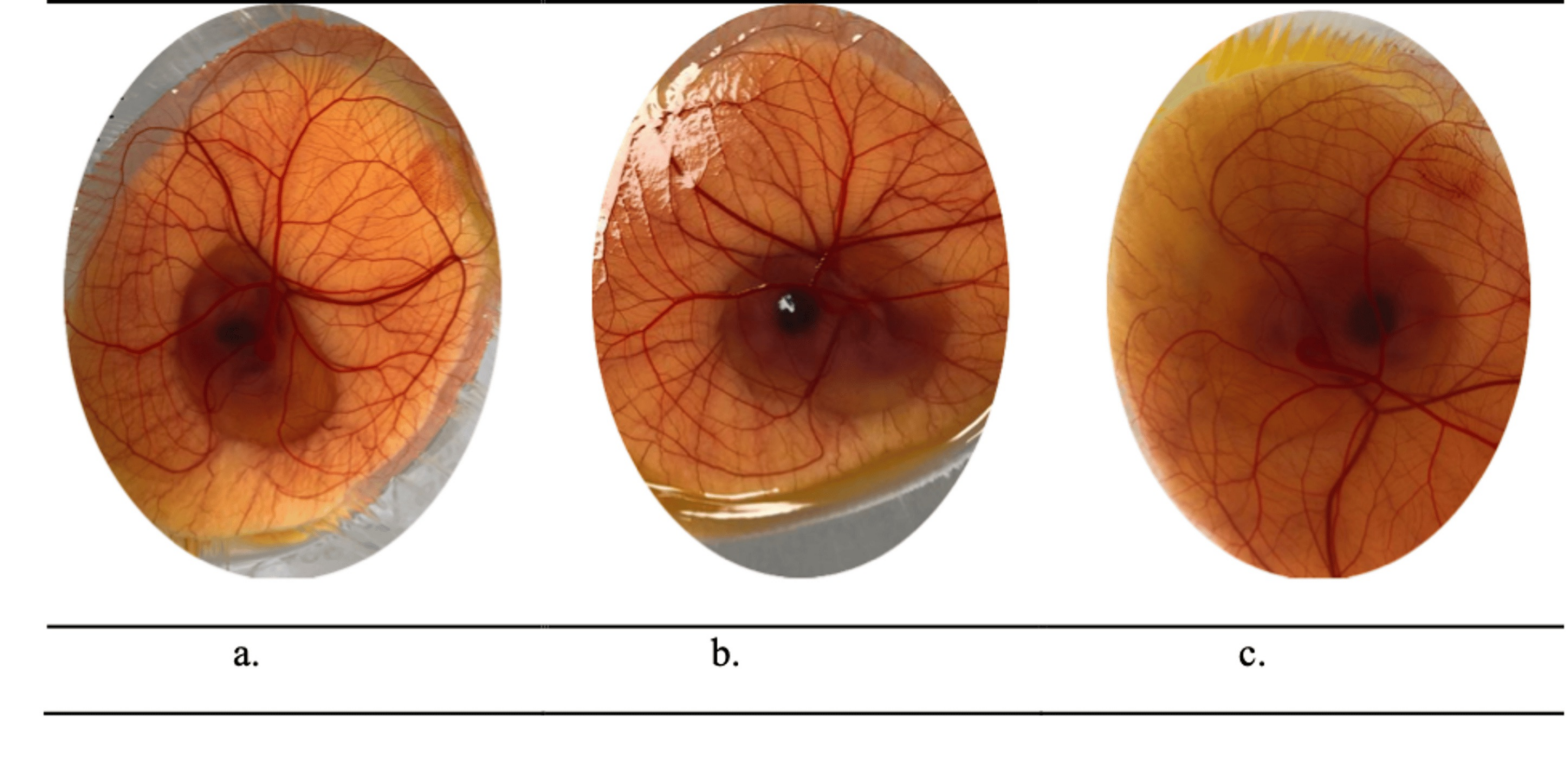
Examination of CAM's surface after five-minute treatment with the test substance: (a) propylene glycol, (b) FB3, and (c) FB1. CAM: Chorioallantoic membrane

The responses of the CAM's surface after the application of test substances and prepared NE formulations are shown in Figure 3.

## Discussion

Optimum ocular bioavailability may require higher concentrations or more frequent dosing of conventional dosage forms like solutions and suspensions, potentially leading to systemic or ocular side effects. The presence of static and dynamic barrier mechanisms leads to a decrease in drug concentration in the anterior chamber. Out of the administered dose, only a small fraction (5%) makes it to the ocular tissues. Moreover, the excessive application of preservatives such as benzalkonium chloride could potentially harm the corneal epithelium or interact with the therapeutic drug. The eye's defense mechanisms, including tear fluid dilution, nasolacrimal drainage, and blinking, eliminate the drug from the eye, causing reduced bioavailability. Due to the eye's anatomy and protective barriers, it is crucial to develop a reliable ocular delivery carrier [17,18].

All prepared NE formulations showed transmittance over 98%, which indicates clear, transparent NE without significant aggregations. Patel et al. emphasized the importance of clear formulation in NE preparations to obtain a one-phase system that is isotropic [19].

By examining conjunctiva damage, the HET-CAM test, which is employed internationally, can determine chemical toxicity as it acts as a vascular mucosal tissue in the human eye as shown in Figure 1. One major drawback of these methods is the lack of a recovery period, which makes it impossible to evaluate the reversibility of ocular tissue lesions [7]. 

Ophthalmic preparations require careful consideration of the safety and tolerability of their ingredients to ensure the comfort and well-being of patients. Triacetin, a non-toxic edible fatty acid, has emerged as a promising candidate for use in ophthalmic formulations due to its favorable properties. NEs, which mainly consist of surfactants, have been observed to cause skin irritations like redness, dryness, and itching by disturbing membrane integrity. Therefore, it is crucial to assess the potential for irritation of surfactants and formulations containing surfactants to guarantee consumer safety [13]. In this study, Tween 80 was found to be practically non-irritating (Table 3). According to other studies, non-ionic surfactants like Tween 80 scored zero in the Draize test, while Tween 20 scored 4, indicating moderate irritation [20]. Non-ionic surfactants are often preferred over other types due to their lower irritation potential. The irritation power of surfactants decreases in the order of cationic > anionic > ampholytic > non-ionic [21].

One of the primary advantages of the oil triacetin is its non-irritant nature. Unlike some traditional ophthalmic excipients, triacetin has been shown to have a minimal impact on ocular tissues, reducing the risk of adverse effects such as irritation, redness, and interference with vision. For this reason, triacetin has been widely used in numerous studies as a safe excipient that does not cause ocular irritation [22,23].

Whereas the cosurfactant propylene glycol caused moderate irritation by causing slight damage to blood vessels when used separately as shown in Figure 3a. Conversely, the presence of propylene glycol did not have the same effect in NE formulations with a high tween 80 content such as F3 and FB3 (Figure 3b), which is recorded to be non-irritant (Table 3). Consequently, the formulations with the highest propylene glycol content (F1 and FB1) showed minimal irritation and slight blood vessel lysis, as shown in Figure 3c, and were recorded to be slightly irritant (Table 3).

This suggests that propylene glycol can be transformed from a moderately irritating substance to a non-irritating one by incorporating it into NE preparations, this result is in agreement with those obtained by Fuime et al., who concluded that undiluted propylene glycol showed only slight irritation to rabbit eyes, despite being an aliphatic alcohol [24].

Propylene glycol has been examined for ophthalmic purposes and confirmed to be non-toxic to rabbit eyes when used in small amounts [25,26]. It was previously reported that undiluted propylene glycol solutions could cause mild redness in the conjunctiva, whereas diluted solutions do not cause irritation [27].

Table 3 demonstrates that the inclusion of BR in the formulations (FB1, FB2, and FB3) did not cause any significant change in irritation scores (p> 0.05) if compared with blank preparations (B1, B2, and B3). Fathalla et al. suggest that, when applied to the eyes of rabbits and rats, BR solutions were determined to be non-irritating [28].

According to the results, it was found that the optimized NE formulations (FB1 and FB3) caused no irritation on the CAM's surface, and the results were comparable to those of the marketed BR eye drop solution.

Limitations of the study

The study successfully developed and evaluated an NE formulation for BR and assessed its irritation potential using the HET-CAM assay. However, some limitations were identified, such as the relatively short time frame available for conducting the experiments, as chick embryos hatch on day 21. Additionally, the experiment required a high number of CAMs, as the double injection method was quite challenging, and a single injection with samples separated by air bubbles might kill the embryos.

## Conclusions

The findings of our study suggest that the ex vivo HET-CAM test is an appropriate method for assessing the irritation potential of NEs, removing the need for further animal testing. By incorporating the HET-CAM test into a series of tests, we can decrease the reliance on mammalian experimentation and minimize harm to animals. Animal experiments may be reduced by utilizing the HET-CAM model and similar alternatives. Additional animal experiments are required for promising compounds due to the limited in vivo biodistribution evaluation. The NE system developed for BR was found to be non-irritating and safe. The results suggest that the prepared NE could offer an effective solution for addressing issues associated with conventional eye drops and delivering BR efficiently to the eyes.
